# Molecular and metabolic insights into purplish leaf coloration through the investigation of two mulberry (*Morus alba*) genotypes

**DOI:** 10.1186/s12870-024-04737-x

**Published:** 2024-01-23

**Authors:** Shusong Li, Yuqing Yang, Jie Yu, Hong Zhou, Zhiwei Hou, Xiling Wang

**Affiliations:** State Key Laboratory of Resource Insects, College of Sericulture, Textile and Biomass Sciences, Southwest University, Tiansheng Road No.2, Chongqing, China

**Keywords:** Mulberry, Leaf coloration, Flavonoid biosynthesis, Transcriptomics, Metabolomics

## Abstract

**Background:**

Leaf coloration in plants, attributed to anthocyanin compounds, plays a crucial role in various physiological functions, and also for pharmaceutical and horticultural uses. However, the molecular mechanisms governing leaf coloration and the physiological significance of anthocyanins in leaves remain poorly understood.

**Results:**

In this study, we investigated leaf color variation in two closely related mulberry genotypes, one with purplish-red young leaves (EP) and another with normal leaf color (EW). We integrated transcriptomic and metabolomic approaches to gain insights into the metabolic and genetic basis of purplish-red leaf development in mulberry. Our results revealed that flavonoid biosynthesis, particularly the accumulation of delphinidin-3-O-glucoside, is a key determinant of leaf color. Additionally, the up-regulation of CHS genes and transcription factors, including MYB family members, likely contributes to the increased flavonoid content in purplish-red leaves.

**Conclusion:**

These findings enhance our understanding of the molecular mechanisms responsible for the purplish coloration observed in mulberry leaves and also offer supporting evidence for the hypothesis that anthocyanins serve a protective function in plant tissues until the processes of light absorption and carbon fixation reach maturity, thereby ensuring a balanced equilibrium between energy capture and utilization.

**Supplementary Information:**

The online version contains supplementary material available at 10.1186/s12870-024-04737-x.

## Introduction

Mulberry leaf is the sole food for silkworms (*Bombyx mori)*, essential in the production of silk yarn. Approximately 90% of the global raw silk production is attributed to mulberry silk, rendering sericulture a highly profitable economic activity, particularly beneficial for rural regions [1]. The quality of the leaves significantly influences cocoon quality, which has a direct impact on the quality of the silk yarn. Notably, the protein found in mulberry leaves serves as the raw material for silk protein, specifically fibroin and sericin, synthesized by the silkworm [2]. Mulberry is also rich in biologically active metabolites, including phenols, flavonoids, alkaloids, terpenoids, amino sugars, and stilbenoids, which exhibit significant potential for antimicrobial, anti-hyperglycemic, anti-hyperlipidemic, antidiabetic, and antioxidative properties [3–5]. Due to these characteristics, mulberry not only used in sericulture but also used in animal feed, making various foodstuffs, and medicine. Taxonomically, mulberry belongs to the Moraceae family, which comprises approximately 52 genera encompassing over 1,400 species [6]s. The *Morus* genus alone includes more than 30 species and 3,000 cultivated varieties distributed worldwide [7]. The diverse mulberry germplasm showcases genotypic and phenotypic variations, prompting distinct physiological, biochemical, and morpho-anatomical responses under varying environmental conditions [8]. Characterized by its deciduous nature, mulberry tree typically exhibits light green or dark green leaves [9, 10]. Only a few genotypes display purplish-red young leaves, which are also identified in many plant species, including maize, tea, maple, lettuce, cotton, poplars. The variation in leaf color often reflects the presence and distribution of essential compounds like chlorophyll, carotenoids, and flavonoids, which contribute to plant physiology and biochemistry [11]. For example, anthocyanin are pigments responsible for red, purple, or blue hues in plants [12]. It is widely accepted that anthocyanin has a shading effect and thereby function as a protector of photosyntem under high light or extreme temperature and other adverse envoriments. Besides, some of the compounds have potential health benefits to human or animals [13].

Flavonoids are a large group of natural substances with variable phenolic structures and are recognized as the pigments responsible for the colors of leaves [14, 15]. Mulberries are known for their richness in flavonoids, including compounds like quercetin 3-(6-malonylglucoside), rutin, isoquercitin, and various prenylated flavonoids [16]. The composition and distribution of flavonoids in mulberry leaves of different resources are very different [17]. The biosynthesis of flavonoids involves a complex metabolic pathway. The core pathway of flavonoid biosynthesis begins with the conversion of phenylalanine, an amino acid, into 4-coumaroyl-CoA. This reaction is catalyzed by phenylalanine ammonia-lyase (*PAL*) and cinnamate-4-hydroxylase (*C4H*), The subsequent steps of the pathway involve the action of enzymes such as chalcone synthase (*CHS*), chalcone isomerase (*CHI*), flavanone 3-hydroxylase (*F3H*), flavonoid 3’-hydroxylase (*F3’H*), dihydroflavonol 4-reductase (*DFR*), and anthocyanidin synthase (*ANS*). These enzymes catalyze reactions that lead to the formation of different classes of flavonoids, including flavones, flavonols, flavanones, anthocyanins, and isoflavonoids.

Pigments present in distinct mulberry variations, exhibiting varying anthocyanin or flavonoid contents, not only contribute to color diversity but also hold immense appeal to medicinal practitioners and pharmaceutical industries due to their bioactive compounds [18]. The “*Morus alba* E” mulberry resources originate from China’s Hubei province. We obtained two lines of E mulberry: one showed pale-green young leaves which is wild-type like (EW) and the other with purplished-red young leaves (EP). Our research aims to identify the genes and metabolites responsible for this coloration and explore their interrelationships through integrated metabolomic and transcriptomic analyses is to unravel the molecular mechanisms underpinning the formation of purplish-red coloration in mulberry leaves.

## Results

### Phenotypic and photosynthetic characteristics of normal mulberry EW and purplish-red leaf mulberry EP

**Fig. 1:**
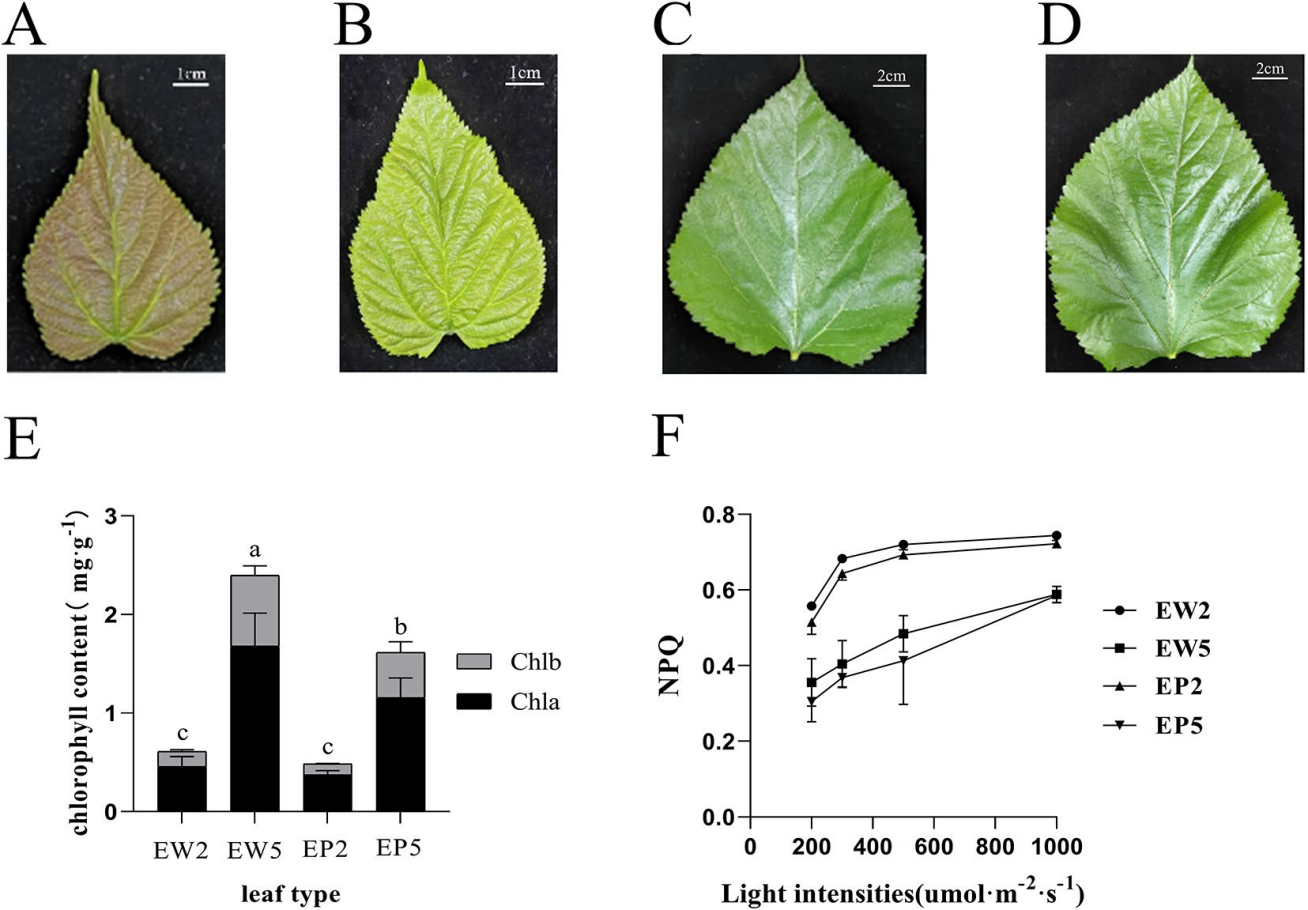
Variance of leaf color, chlorophyll content and non-photoquenching (NPQ) of EP and EW. (**A–D**) Phenotype of the second leaf for EP (EP2, A) and EW (EW2, B) and the fifth leaf of EP (EP5, C) and EW (EW5, D). (**E**) Chlorophyll content in leaves. (**F**) The NPQ value of leaves exposure to different light intensities. Statistical significance within the figure is denoted by alphabetical markers (a, b, c, d), where different letters indicate statistically significant differences among groups or conditions. Shared letters imply no statistically significant difference between those particular groups or conditions. Experiments were conducted in triplicate

EW and EP were grafted onto the same stump but exhibited different colors during their early development. Young leaves of EW appeared yellow-green, while EP displayed a purplish-red color (Fig. 1A, B). However, as they reached the fifth leaf stage and fully opened, the leaves of EP turned dark green, and no phenotypic differences were observed between EP and EW (Fig. 1C, D). To investigate this color difference, we measured the chlorophyll content in the second leaf. Our results indicated that the contents of chlorophyll a and b in the second leaf of EP (EP2) were significantly lower than in the corresponding leaves of EW (EW2, Fig. 1E). Additionally, we analyzed their photosynthesis capacities and found no significant differences in key parameters such as the net assimilation rate of CO_2_ (A), stomatal conductance (gs), intercellular carbon dioxide concentration (Ci), and leaf transpiration rate (E) between EP2 and EW2 (Fig. S1A-D). Furthermore, the maximum quantum efficiency of PSII photochemistry (Fv /Fm) showed no significant difference, indicating that PSII efficiency was similar in the two mulberry lines. Similarly, no differences were observed in Fm/Fo, Fm/Fo, Mo, and Fv’/Fm’ between EP2 and EW2 leaves. However, the non-photochemical quenching (NPQ) of EP2 leaves was significantly lower than that of EW2 leaves (Table 1; Fig. 1F).

**Table 1:** Chloropyll fluorescence of the second leaf of EP and EW. Asterisk stands for asterisk stands for *p* < 0.05 for EW2 compared to EP2. Each experiment had three biological replications

Type name	Fv/Fo	Fm/Fo	FV/Fo	Mo	Sm	NPQ
EW2	2.85	3.85	0.74	0.79	377.89	2.57
EP2	2.68	3.68	0.73	0.92	390.87	2.02*

### Overview of the metabolic profiles of the mulberry leaves from EP and EW lines

To gain comprehensive insights into the differential metabolites between the EP and EW genotypes, we conducted a broad-target metabolomics analysis of mulberry leaf metabolites. We constructed an MS2 spectral tag library comprising 9,300 molecular features and then performed liquid chromatography tandem mass spectrometry (LC-MS/MS) on mulberry leaf extracts. In total, we identified 621 metabolites in mulberry leaves, spanning more than 35 classes, including carbohydrates, amino acids and derivatives, fatty acyls, vitamins, and secondary metabolites. Detailed imformation were included in Table S1.

**Fig. 2:**
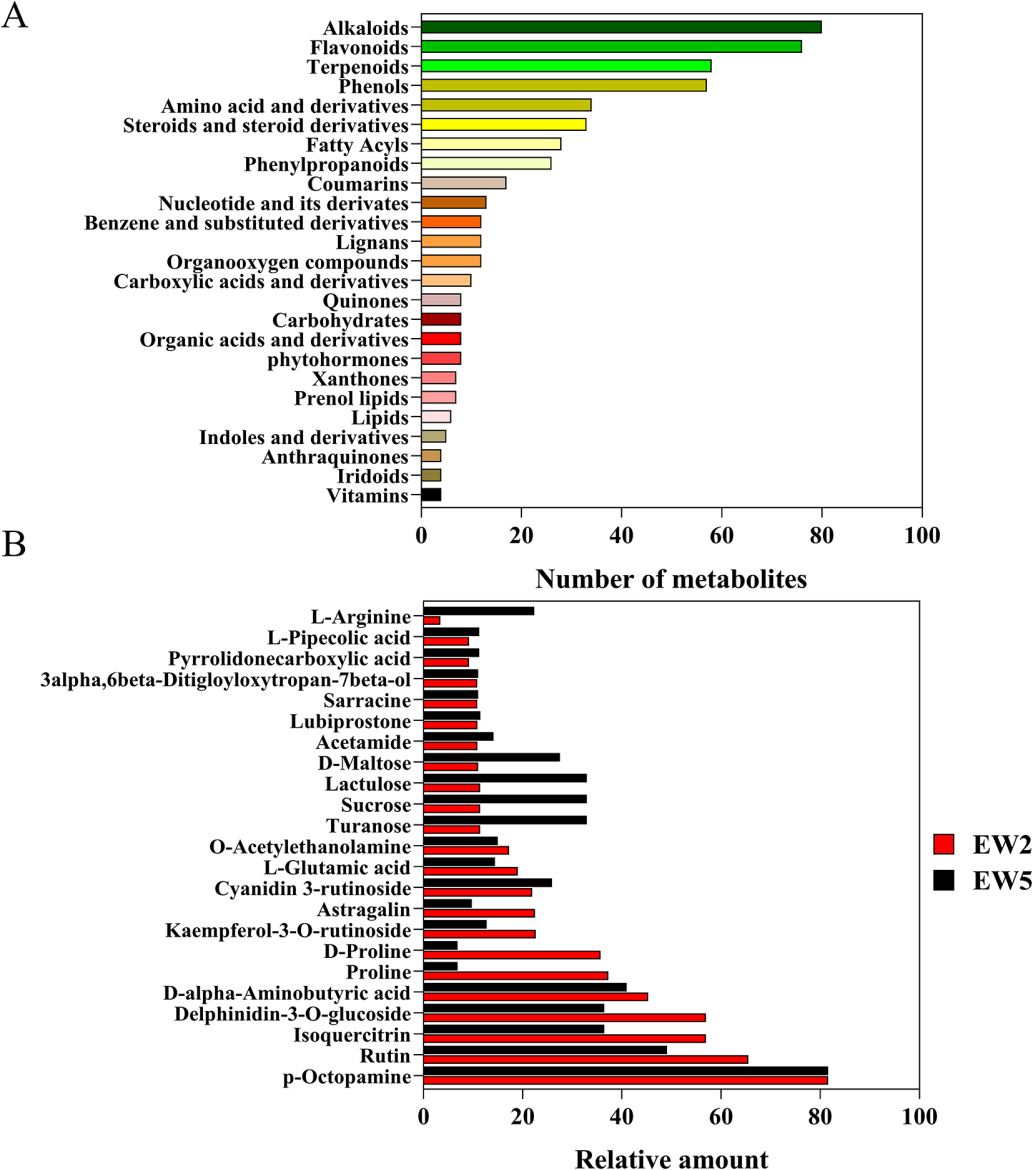
Overview of metabolites detected in mulberry leaves. (**A**) Classification and distribution of metabolites detected in mulberry leaves. (**B**) Relative amount of metabolites in the second leaf (EW2) and fifith leaf (EW5)

The six most abundant classes of metabolites identified were alkaloids (80 compounds), flavonoids (77 compounds), phenols (59 compounds), terpenoids (47 compounds), amino acids and their derivatives (35 compounds), and steroids and steroid derivatives (33 compounds) (Fig. 2A). Specific metabolites like p-octopamine, isoquercitrin, delphinidin-3-O-glucoside, and astragalin were found to have higher contents than other compounds in mulberry leaves. Notably, the content of certain metabolites, including rutin, isoquercitrin, delphinidin-3-glucoside, D-alpha-aminobutyric acid, proline, D-proline, kaempferol-3-O-rutinoside, and astragalin, in the second leaf was significantly higher than that in the fifth leaf. Conversely, the content of metabolites such as turanose, sucrose, lactulose, D-maltose, and L-arginine in the fifth leaf was significantly higher than that in the second leaf (Fig. 2B).

### Metabolite profiling and characterization of EP and EW leaves

**Fig. 3:**
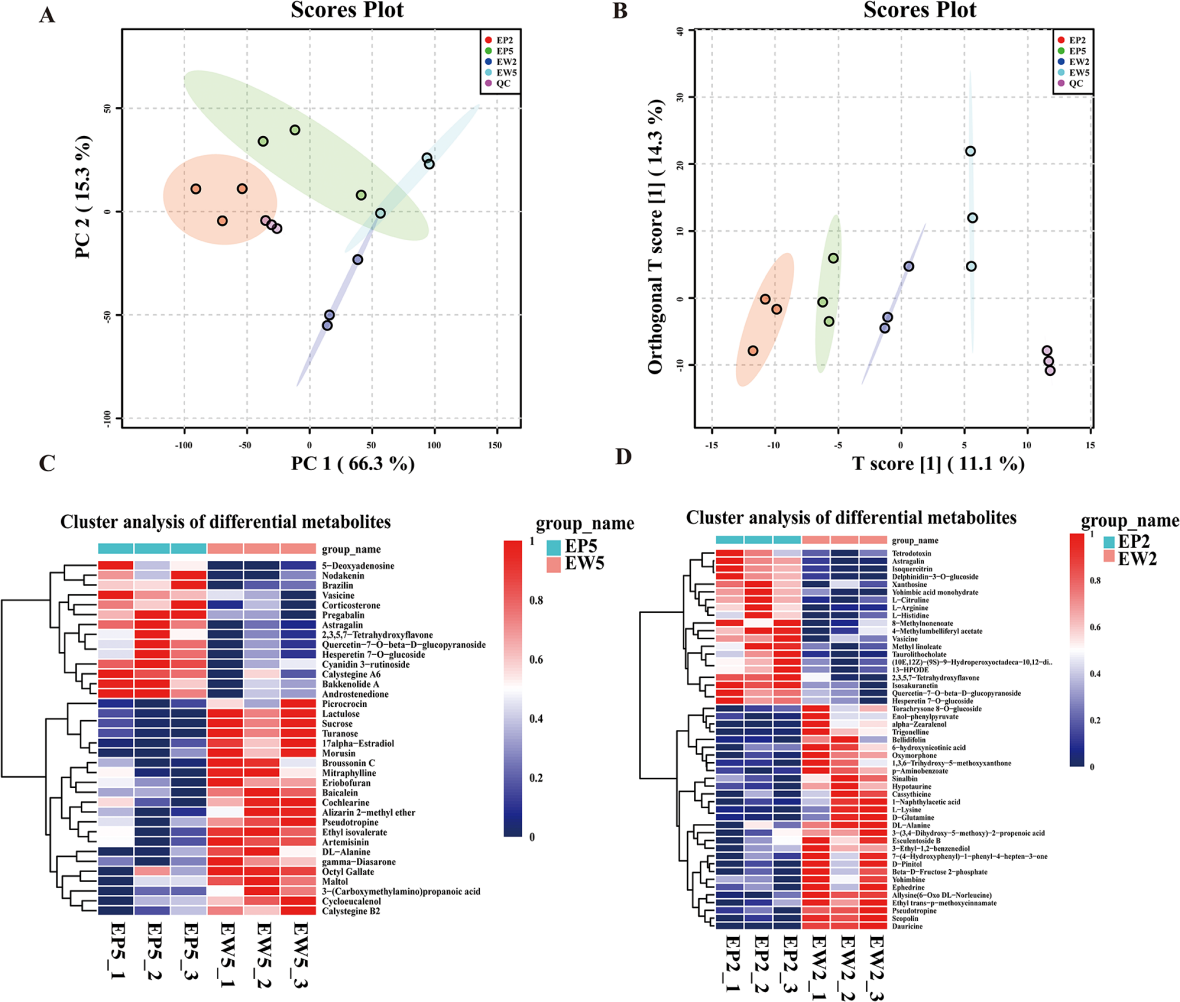
Metabolic profiling and analysis in the second and fifth mulberry leaves. (**A**) Principal Component Analysis (PCA) of metabolic profiles in mulberry leaves. (**B**) Score plots generated from Orthogonal Partial Least Squares Discriminant Analysis (OPLS-DA) of metabolic profiles in mulberry leaves. (**C–D**) Cluster analysis of differential metabolites in the second (**C**) and fifith leaves (**D**) of EP and EW. QC, quanlity control samples. After the sample extraction, all samples of equal volume are taken and then mixed

**Fig. 4:**
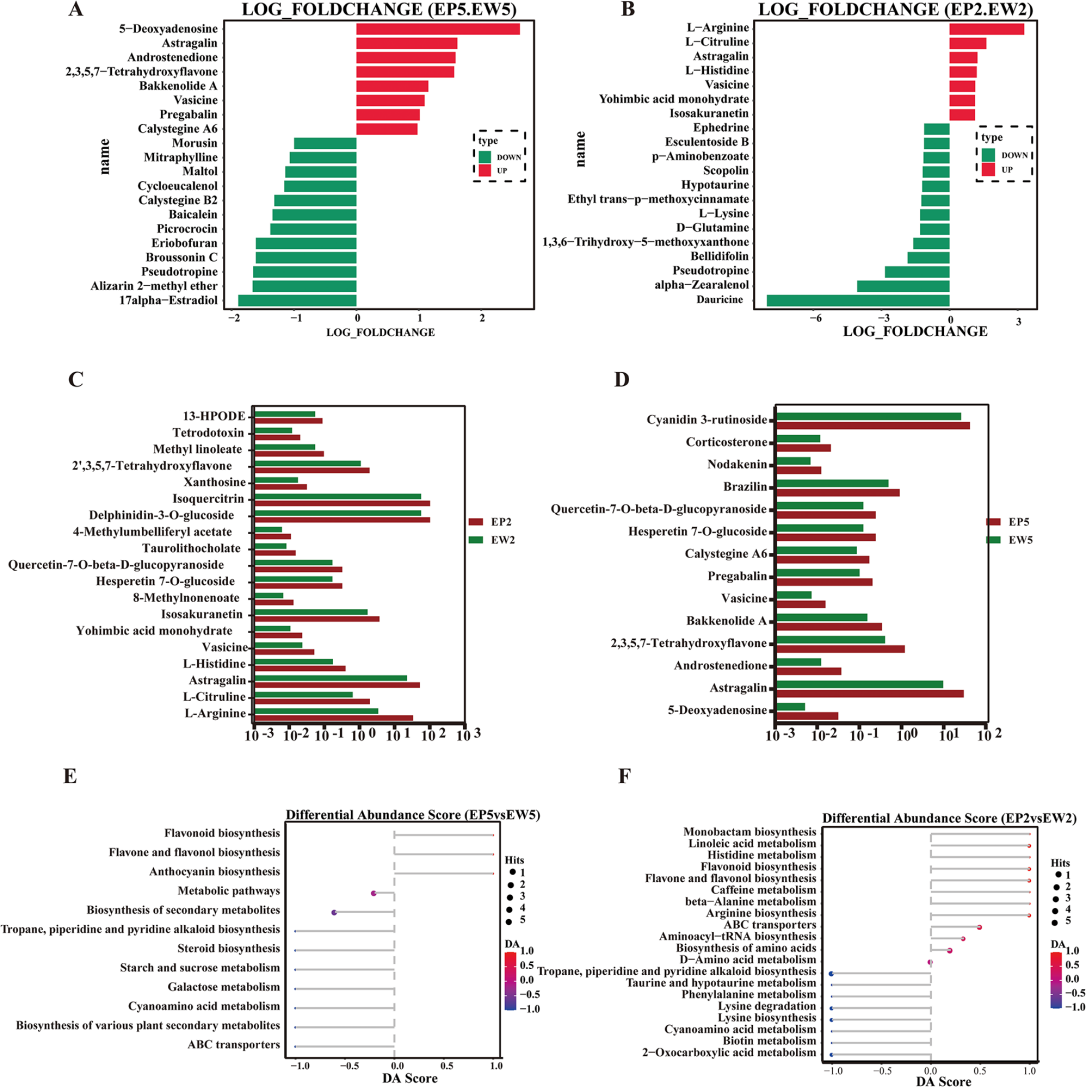
Differential expressed metabolites (DEMs) in mulberry leaves. (**A, B**) Histogram of DEMs in EP5 VS EW5 and EP2 VS EW2 mulberry leaves. (**C, D**) Relative abundance of DEMs in EP and EW mulberry leaves. (**E, F**) Differential abundance score map of differential metabolic pathways in EP5 VS EW5 and EP2 VS EW2 mulberry leaves. EP2 and EW2 represent the second leaf of EP and EW, while EP5 and EW5 represent the fifth leaf of EP and EW

All detected metabolites from EP and EW were subjected to Principal Component Analysis (PCA), resulting in clear separation into four distinct groups (Fig. 3A). This separation was further confirmed by Orthogonal Partial Least Squares Discriminant Analysis (OPLS-DA) plot (Fig. 3B) and heatmap analysis (Fig. 3C and D), demonstrating significant differences between EP and EW metabolite profiles. Using specific criteria (|log2(Fold Change)| ≥ 1 and Padj ≤ 0.05), we identified 49 differentially expressed metabolites (DEMs) between EP2 and EW2, including 29 up-regulated and 20 down-regulated metabolites. These DEMs comprised mainly 5 amino acids and derivatives, 7 flavonoids, 9 alkaloids, and other compounds. Notably, L-Arginine exhibited the most significant up-regulation, while dauricine showed the most significant down-regulation (Fig. 4A). Between EP5 and EW5, we identified 36 DEMs, including 14 up-regulated and 22 down-regulated metabolites, with 9 flavonoids, 8 alkaloids, and other compounds (Fig. 4B). Remarkably, certain metabolites, including astragalin, vasicine, 2,3,5,7-tetrahydroxyflavone, hesperetin-7-O-glucoside, and quercetin-7-O-beta-D-glucopyranoside, were identified as co-DEMs among the up-regulated metabolites (Fig. 4C, D). However, their contents were higher in EP than in EW. Correlation analysis of the top 17 DEMs with the highest difference significance revealed strong positive or negative correlation coefficients, indicating a high correlation and significant differences among these DEMs (Fig. 4E, F).

To better understand the functional implications of the marked differences between EP and EW metabolites, we conducted KEGG enrichment analyses. The results indicated that the DEMs in EP2 and EW2 were primarily associated with pathways such as linoleic acid metabolism (ath00591), flavonoid biosynthesis (ath00941), flavone and flavonol biosynthesis (ath00944), arginine biosynthesis (ath00220), ABC transporters (ath02010), aminoacyl − tRNA biosynthesis (ath00970), tropane, piperidine and pyridine alkaloid biosynthesis (ath00960), lysine biosynthesis (ath00300), lysine degradation (ath00310), 2-Oxocarboxylic acid metabolism (ath01210), and cyanoamino acid metabolism (ath00460). Among them, flavonoids biosynthesis (ath00941), flavone and flavonol biosynthesis (ath00944), tropane, piperidine and pyridine alkaloid biosynthesis (ath00960), and cyanoamino acid metabolism (ath00460) were also enriched in EP5 and EW5 but with a reduced density of enrichment (Table 2). These results suggest that the differences between the purplish-red leaves and green leaves are mainly attributed to the biosynthesis of flavonoids, piperidine and pyridine alkaloids, and amino acids and derivatives.

**Table 2:** Significantly enriched KEGG pathways between EP and EW

	NO.	Pathway	Pathway ID	DEGs with pathway annotation	All genes with pathway annotation	p-value
EP5vs EW5	1	Flavonoid biosynthesis	ath00941	18	22	0.0022
	2	Monoterpenoid biosynthesis	ath00902	7	8	0.041
	3	Valine, leucine and isoleucine biosynthesis	ath00290	13	22	0.048
	4	DNA replication	ath03030	30	50	0.00021
	5	Ribosome	ath03010	145	364	0.0043
	6	Circadian rhythm - plant	ath04712	24	36	0.0054
	7	Base excision repair	ath03410	24	43	0.015
	8	Mismatch repair	ath03430	22	39	0.017
	9	Pyruvate metabolism	ath00620	39	86	0.029
	10	Fatty acid metabolism	ath01212	32	69	0.036
	11	Fatty acid biosynthesis	ath00061	22	43	0.037
	12	Zeatin biosynthesis	ath00908	17	32	0.049
EP2 vs. EW2	1	Stilbenoid, diarylheptanoid and gingerol biosynthesis	ath00945	3	7	0.0014
	2	Butanoate metabolism	ath00650	4	19	0.0019
	3	Flavonoid biosynthesis	ath00941	4	22	0.0031
	4	C5-Branched dibasic acid metabolism	ath00660	2	7	0.018
	5	Glycerolipid metabolism	ath00561	5	60	0.019
	6	Biosynthesis of secondary metabolites	ath01110	38	1107	0.021
	7	Pantothenate and CoA biosynthesis	ath00770	3	28	0.036
	8	Porphyrin and chlorophyll metabolism	ath00860	4	53	0.046

### Identification and functional annotation of differentially expressed genes between EP and EW

**Fig. 5:**
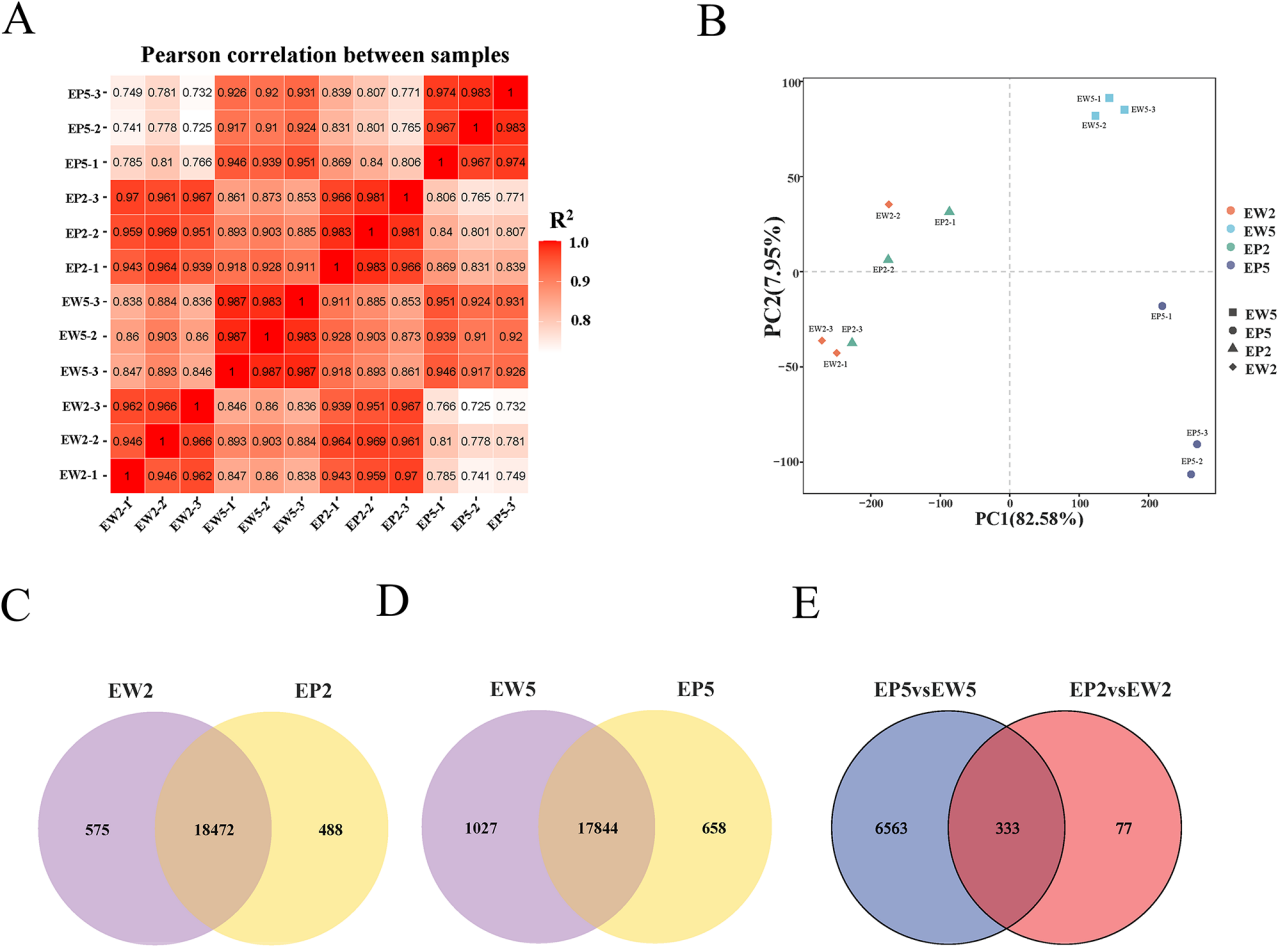
Comparative analysis of gene expression in mulberry leaves. (**A**) Expression quantity correlation thermogram in mulberry leaves. (**B**) Principal component analysis (PCA) of mulberry leaf transcriptome. (**C–E**) Venn diagrams showing significantly differently expressed genes between EW2 vs. EP2, EW5 vs. EP5, and EP5 vs. EW5 as well as EP2 vs. EW2. EP2 and EW2 represent the second leaf of EP and EW, while EP5 and EW5 represent the fifth leaf of EP and EW

To elucidate the molecular mechanisms underlying the up or down-regulation of metabolites in EP, we performed RNA-Seq analysis and constructed six RNA-Seq libraries for paired-end sequencing. We obtained a total of 7.1 Gigabase (Gbp) clean reads from EW and 6.49 Gbp of clean base reads from EP, with high quality indicated by Q30 values over 93.54% and Q20 values over 91.6% and 82.87%, respectively. The mapping rate and unique mapped rate were both higher than 91.6% and 82.87%, respectively. High reproducibility was confirmed by calculating the correlation coefficients between the three biological replicates, all of which exceeded 0.93 (Fig. 5A). Principal component analysis (PCA) of the transcriptome data revealed that PC1 (82.58%) and PC2 (7.95%) explained 90.45% of the variation in the transcriptome profile. The samples were divided into three groups along PC1, with EP2 and EW2 samples mixing together and clearly separating from EW5 and EP5 samples. EW5 and EP5 samples were very close to each other among the three replicates, except for EP5-1 (Fig. 5B). In total, 22,090 unique genes were detected among the four mulberry leaf samples. Using specific criteria (|log2 (Fold Change)| ≥ 1 and Padj ≤ 0.05), we identified differentially expressed genes (DEGs) between EP and EW.

In general, we detected 18,960 genes expressed in EP2 and 19,047 genes expressed in EW2, with 18,472 genes co-expressed between the two genotypes (Fig. 5C). Similarly, we found 18,502 expressed genes in EP5 and 18,916 expressed genes in EW5, with 17,844 co-expressed genes between them (Fig. 5D). Identified gene names were listed in Table S2. Comparing EP2 vs. EW2, we identified a total of 410 differentially expressed genes (DEGs), including 161 up-regulated genes and 249 down-regulated genes. Besides, 6,896 DEGs were identified in EP5 vs. EW5, comprising 3,538 up-regulated genes and 3,358 down-regulated genes. Identified DEGs were listed in Table S2. Venn diagram analysis indicated that 333 DEGs were common in EP2 vs. EW2 and EP5 vs. EW5 (Fig. 5E). These genes differed between the EP and EW mulberry leaves, some of which may be responsible for the purplish-red leaf formation

### GO and KEGG functional enrichment analysis of differentially expressed genes

**Fig. 6:**
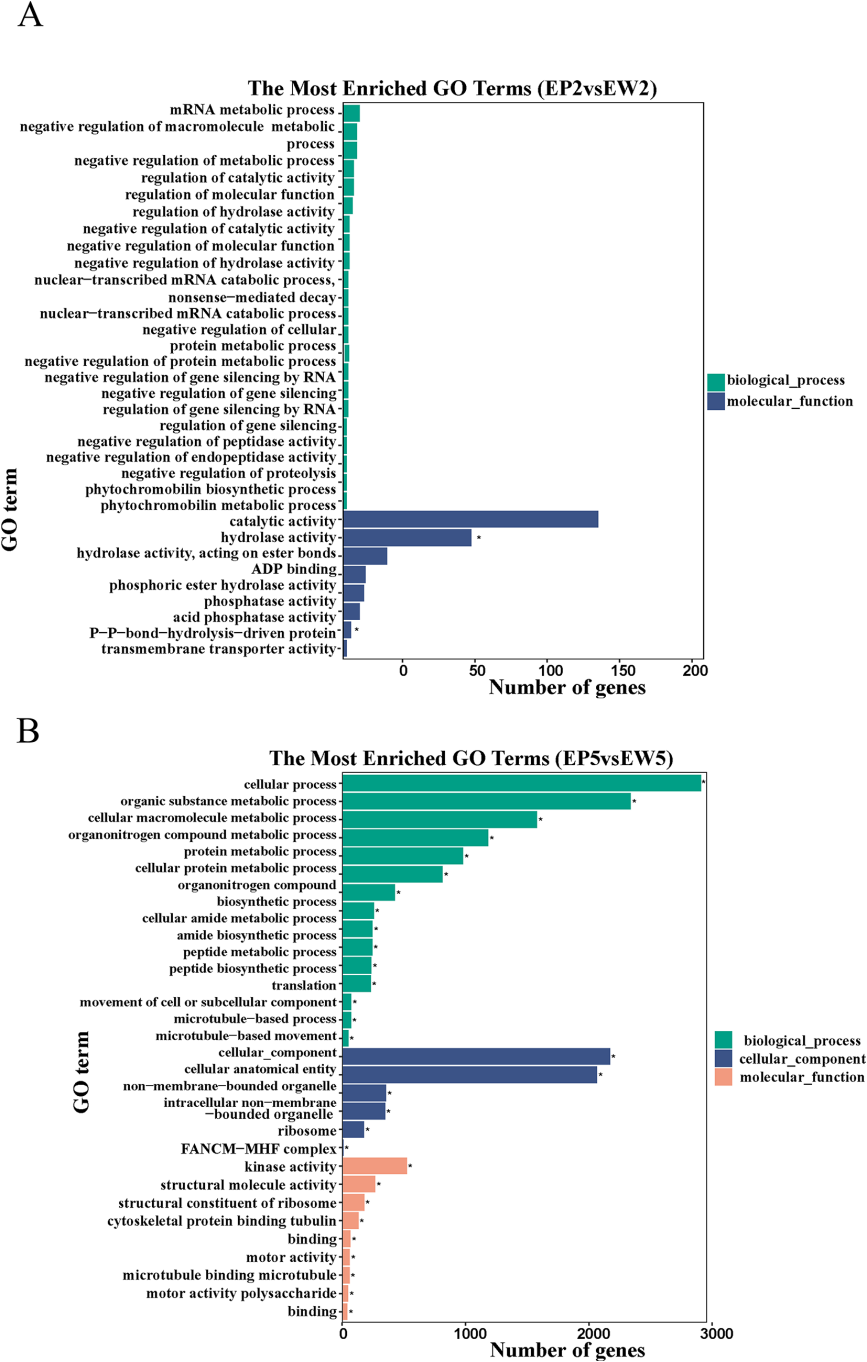
Analysis of GO enrichment of differentially expressed genes in EP2 vs. EW2 (**A**) and EP5 vs. EW5 (**B**)

GO functional enrichment analysis classified the DEGs into three categories: molecular function, cellular component, and biological process. For EP2 vs. EW2, the top 30 GO terms significantly enriched were predominantly related to catalytic activity (176) and hydrolase activity (88). For EP5 vs. EW5, the top 30 significantly enriched GO terms spanned three categories, with most of the enriched terms associated with cellular processes, organic substance metabolic processes, cellular macromolecule metabolic processes, organonitrogen compound metabolic processes, cellular components, cellular anatomical entities, and kinase activity (Fig. 6A).

Additionally, KEGG enrichment analyses of the DEGs were conducted. For the comparison between EP2 and EW2, the pathways ‘stilbenoid, diarylheptanoid, and gingerol biosynthesis’ (ath00945), ‘butanoate metabolism’ (ath00650), ‘flavonoid biosynthesis’ (ath00941), ‘C5-Branched dibasic acid metabolism’ (ath00660), ‘glycerolipid metabolism’ (ath00561), ‘biosynthesis of secondary metabolites’ (ath01110), ‘Pantothenate and CoA biosynthesis’ (ath00770), and ‘porphyrin and chlorophyll metabolism’ (ath00860) were significantly enriched (Fig. 6B). For EP5 vs. EW5, KEGG analysis revealed significant changes in pathways such as DNA replication (ath03030), flavonoid biosynthesis (ath00941), Ribosome (ath03010), Circadian rhythm – plant (ath04712), Base excision repair (ath03410), Mismatch repair (ath03430), Pyruvate metabolism (ath00620), Fatty acid metabolism (ath01212), Fatty acid biosynthesis (ath00061), Monoterpenoid biosynthesis (ath00902), Valine, leucine, and isoleucine biosynthesis (ath00290), and Zeatin biosynthesis (ath00908) (Fig. 6B). These results suggest that pathways related to porphyrin and chlorophyll metabolism, as well as secondary metabolites like flavonoid biosynthesis, play significant roles in the color change of leaves.

### Transcription factor analysis of differentially expressed genes

**Fig. 7:**
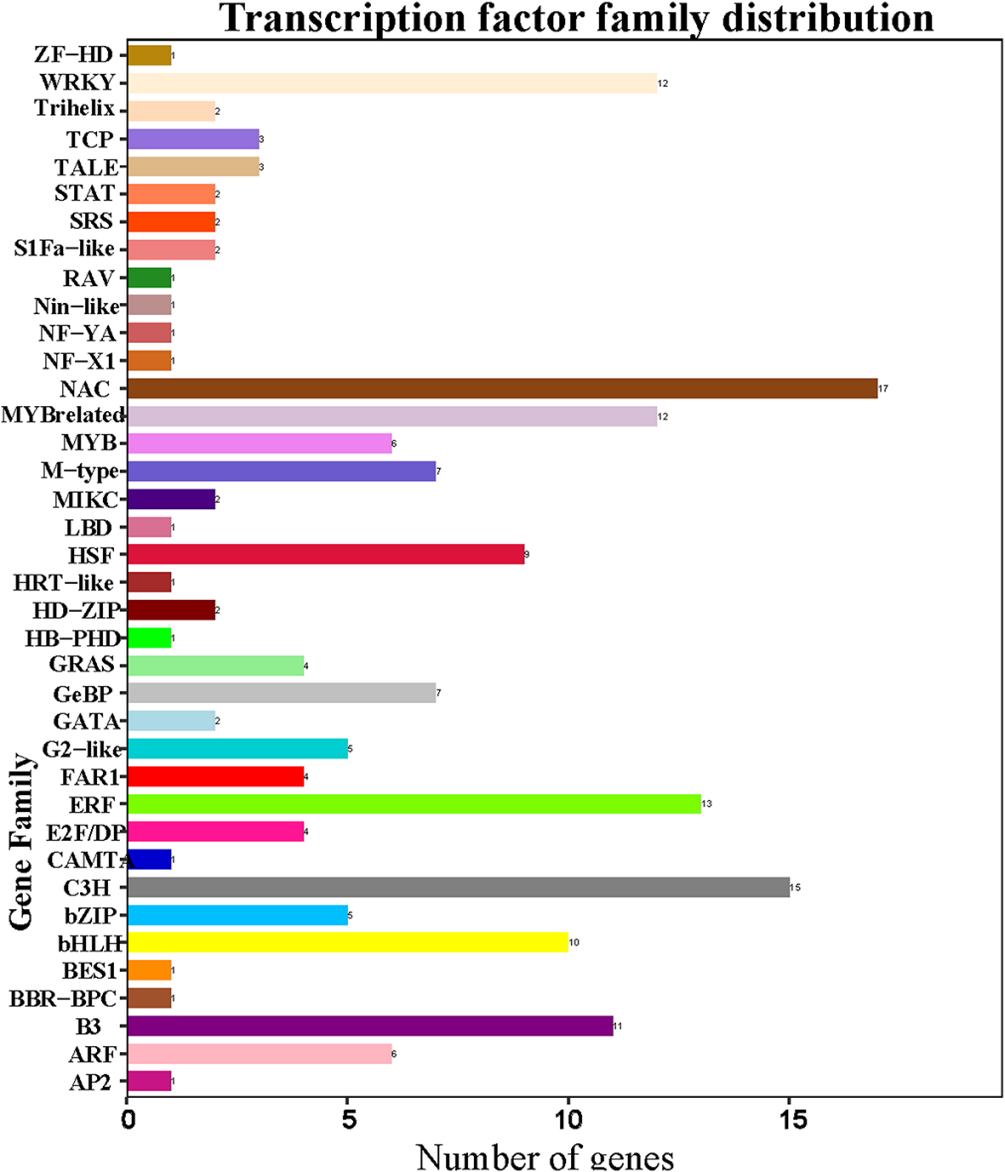
Transcription factor family distribution of differently expressed genes in EP2 compared to EW2

In our transcriptomic analysis between EP2 and EW2, we identified a total of 179 differentially expressed genes (DEGs) annotated as transcription factors (TFs) by Plant Transcription Factor Database [19]. These TFs belonged to various families, with prominent representation from NAC (17), C3H (15), ERF (13), MYB-related (12), and WRKY (12) families (Fig. 7). The detailed gene name, TF-ID and predicted gene family were listed in Table S3. Notably, the MYB family has been extensively associated with the regulation of secondary metabolism, particularly flavonoid biosynthesis [20]. Several research investigations indicate that the regulation of anthocyanin biosynthesis genes is intricately governed by transcription factors, notably, by a complex termed the MYB-bHLH-WD40 (MBW) complex, which comprises MYB, basic helix-loop-helix (bHLH), and WD40 repeat families [21]. In this study, six MYB TFs (malb00000676, malb00005066, malb00005100, malb00020549, malb00021415, Novel01160) and 12 MYB related TFs were identified as DEGs between EP2 vs. EW2 and could be regulatory genes for flavonoid biosynthesis. Among them, malb00000676 is the homolog gene to Arabidopsis AT4G37260 (MYB73, Figure S2), which has been proved to be associated with the anthocyanin under low temperature conditions [22].

Furthermore, among the observed changes in gene expression between EP2 and EW2, 10 predicted bHLH TFs showed significant alterations. However, despite these changes, their sequence similarity to known bHLH TFs was not identified through annotations in the Non-redundant Protein Sequence Database in GenBank or by comparison with the model plant Arabidopsis. WRKY proteins form a substantial TF family known for their conserved WRKY DNA binding domain [23]. They have been implicated in various roles in plant growth and development [24]. Specific WRKY proteins like TTG2 and PH3 have been recognized for their influence on flavonoid biosynthesis by interacting with the WBM complex in Arabidopsis and Petunia, respectively [25, 26]. Additionally, examples from Brassica napus WRKY41-1 and Arabidopsis WRKY75 demonstrate their roles as repressors in regulating anthocyanin biosynthesis [27]. In our study, we observed differential expression of malb00000833 in EP2 compared to EW2. Through our investigation, we confirmed that malb00000833 is a homolog to MdWRKY40 in apple (*Malus domestica*, Fig S3). Interestingly, prior research has highlighted this gene as a significant regulator in wound-induced anthocyanin biosynthesis [28].

### Combined transcriptome and metabolome analysis of key metabolic pathways between EP and EW

To comprehensively understand the functions and regulatory mechanisms of biomolecules responsible for leaf color changes between EP and EW, we conducted correlation analysis based on the Pearson correlation coefficient. The results of correlation analysis between DEMs and DEGs showed a predominantly negative correlation, and the same differentially expressed metabolite exhibited the same correlation direction (positive/negative) with different DEGs.

**Fig. 8:**
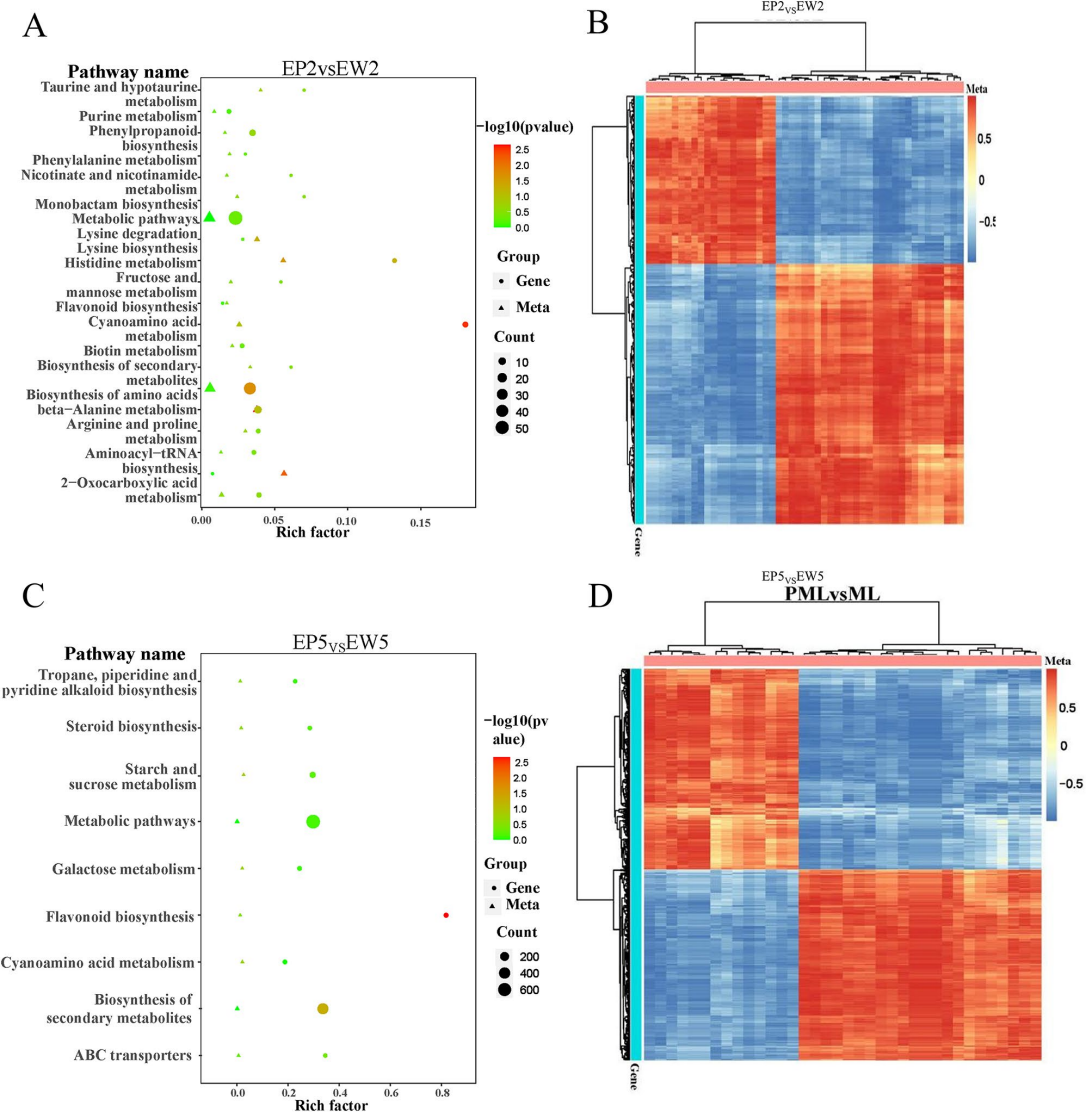
Integrated transcriptome and metabolic analysis in mulberry leaves. (**A–C**) Bubble Chart illustrating the KEGG pathways jointly enriched from Differentially Expressed Genes (DEGs) and Differential Metabolites (DMs) in EP5 VS EW5 and EP2 VS EW2. (**B–D**) Cluster Heatmap representing the profiles of DEGs and DMs in EP5 VS EW5 and EP2 VS EW2. EP2 (purplish-red leaf) and EW2 are the second leaf of EP and EW and EP5 and EW5 are the fifith leaf of EP and EW

Subsequently, we performed KEGG enrichment analysis to identify key metabolic pathways and genes associated with the leaf color changes in EP and EW [29]. Between EP2 and EW2, 20 common KEGG enrichment pathways were identified for both DEMs and DEGs. The pathways with high confidence included flavonoid biosynthesis, aminoacyl-tRNA biosynthesis, and biosynthesis of secondary metabolites (Fig. 8A, B). Similarly, between EP5 and EW5, nine common KEGG enrichment pathways were identified for both DEMs and DEGs, with flavonoid biosynthesis and biosynthesis of secondary metabolites being the pathways of high confidence (Fig. 8C, D). These findings suggest that these pathways are likely involved in mulberry leaf color change and may play essential roles in the regulation of the observed differences between EP and EW.

**Fig. 9:**
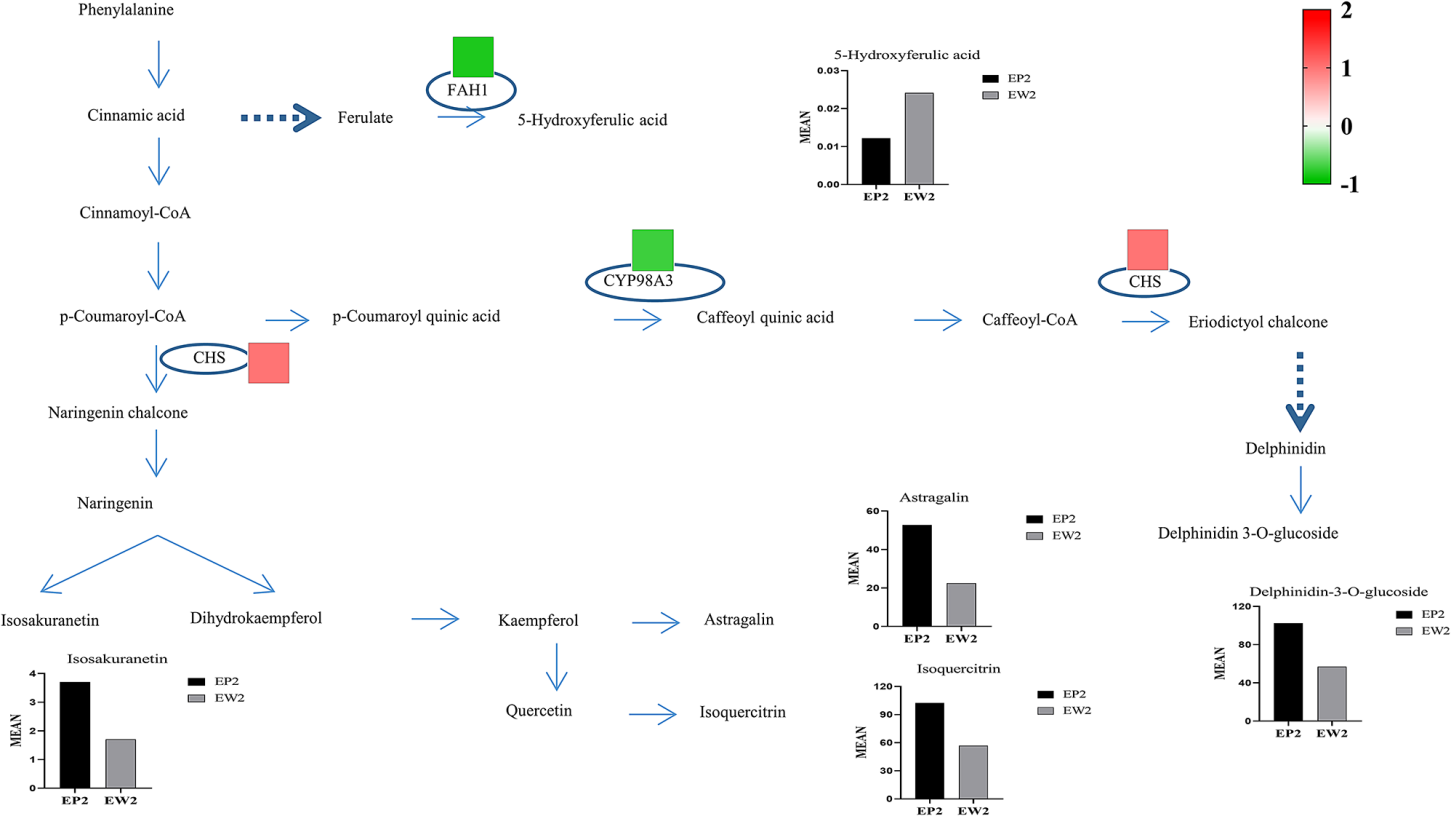
Differentially expressed genes (DEGs) and metabolites (DEMs) in flavonoid biosynthesis. Histograms showed the relative expression of DEMs. Each colored cell represents the average log2(RPKM) value of gene expression EP2/EW2 according to the color scale

Genes involved in flavonoid synthesis were identified. Using KEGG and MapMan annotations (https://mapman.gabipd.org/mapman), we identified 27 DEGs associated with flavonoid biosynthesis in EP2 compared to EW2 (refer to Table S4). The expression patterns of these genes across various leaf samples were visualized in the heatmap (Fig S4). Our metabolome analyses revealed some key compounds that were up-regulated in EP2 compared with EW2, such as delphinidin-3-O-glucoside, astragalin, cyanidin 3-rutinoside, and kaempferol-3-O-rutinoside. However, the expression of their directly catalytic genes did not significantly change. We observed a high expression of chalcone synthase (CHS) and two down-regulated genes, including FAH1 and CYP98A3 that were potentially correlated with the biosynthesis of delphinidin-3-O-glucoside (Fig. 9). *CHS* is widely regarded as a key enzyme required for the accumulation of purple anthocyanins in leaves and stems, suggesting its involvement in the elevated purple component, delphinidin 3-O-glucoside, in EP2.

## Discussion

Purplish-red coloration in various plant tissues, such as flowers and fruits, is often linked to the biosynthesis and regulation of anthocyanin compounds [30]. However, there has been relatively limited research on the role of anthocyanins in leaf coloration, and their physiological functions in vegetative organs remain unclear. In our study, we investigated two closely related mulberry genotypes, EP with purplish-red young leaves and EW with normal leaf color (Fig. 1). Although their photosynthesis capacities were similar, we observed reduced non-photochemical quenching (NPQ) in purplish-red leaves (Fig. 1F). While the presence of anthocyanins leading to coloration in juvenile leaves is common in tree species near the equator [31–33], the molecular mechanisms behind the development of purplish-red leaves have remained poorly understood. In this study, we conducted a comprehensive analysis of EP and EW leaves, integrating transcriptomic and metabolomic approaches to explore the molecular mechanisms underlying the purplish-red phenotype in mulberry genotypes.

Through metabolomic analyses, we identified a wide range of metabolites in mulberry leaves, including carbohydrates, amino acids and derivatives, fatty acyls, vitamins, and secondary metabolites. Notably, flavonoids, alkaloids, phenols, terpenoids, and amino sugars were among the most abundant metabolites in both genotypes. These compounds have been previously recognized for their biologically active properties, including antimicrobial, antihyperglycemic, antihyperlipidemic, antidiabetic, and antioxidant potentials. This suggests that mulberry leaves are not only suitable for silkworm feed but also hold potential value for various applications, such as feed additives and medicinal uses [34]. Among the identified metabolites, p-octopamine, isoquercitrin, delphinidin-3-O-glucoside, astragalin were found to be dominant compounds in mulberry leaves (Fig. 2). These compounds have diverse roles, with p-octopamine potentially influencing insect-plant interactions or biotic stress responses [35], isoquercitrin and astragalin having antioxidant and anti-inflammatory properties [36], and delphinidin-3-O-glucoside belonging to anthocyanins, which are responsible for the red, purple, and blue pigments in various plant tissues, including leaves, flowers, and fruits [37–39]. The abundant presence of those components in mulberry leaves underscores their potential biological significance.

Metabolic profilings of different mulberry resources were previous analyzed [17, 40], but limited information is available regarding metabolite changes in different leaf stages. In our study, we investigated metabolic changes in the second and fifth leaves. The higher abundance of rutin, isoquercitrin, delphinidin-3-glucoside, D-alpha-aminobutyric acid, proline, D-proline, kaempferol-3-O-rutinoside, and astragalin in the second leaf suggests their crucial roles during early leaf development. For example, rutin and isoquercitrin, both flavonoid glycosides, are known for their antioxidant and anti-inflammatory properties, and their abundance in the second leaf might contribute to the plant’s defense against oxidative stress and environmental challenges [41]. Delphinidin-3-glucoside, as an anthocyanin, is responsible for red or purple coloration in plants. In contrast, the higher levels of turanose, sucrose, lactulose, D-maltose, and L-arginine in the fifth leaf suggest that they might have specific functions during the later stages of leaf development or senescence. For instance, sucrose is a major transport sugar in plants, and its increased levels in the fifth leaf compared to the second leaf could be related to elevated sugar production, carbohydrate storage, and redistribution as the leaf matures [42]. The differential accumulation of these metabolites between the second and fifth leaves likely reflects dynamic metabolic changes essential for optimizing resource allocation, stress tolerance, and overall plant fitness. Further research is needed to elucidate the underlying molecular and physiological mechanisms governing the differential accumulation of these metabolites in different leaf stages, which could provide valuable insights into plant growth and adaptation strategies, as well as potential applications in agriculture and crop improvement.

In plants, the coloration of leaves, flowers, and fruits is often attributed to the presence and combination of various pigments, including chlorophylls, carotenoids, and anthocyanins [13]. Chlorophylls impart green coloration, carotenoids contribute to yellow, orange, and red hues, while anthocyanins are responsible for red, purple, and blue colors [43]. The presence of specific compounds, such as anthocyanins and flavonoids, in plants can lead to the development of purplish-red leaves. Anthocyanins, in particular, are well-known for providing red, purple, or blue coloration to various plant tissues, including leaves [44]. Our results revealed significant differences in the metabolic and gene expression profiles between EP and EW. Specifically, flavonoids were the most enriched differentially expressed metabolites (DEMs), and flavonoid biosynthetic genes were the most enriched differentially expressed genes (DEGs), indicating substantial metabolic and gene expression differences between the two variants in the composition and synthetic pathway of flavonoids. These likely play an essential role in determining leaf color. Delphinidin-3-O-glucoside, astragalin, cyanidin 3-rutinoside, and kaempferol-3-O-rutinoside were found at high levels in the second leaves compared to other compounds and were significantly higher in EP2 than in EW2. These dominant flavonoids may have a combined effect on leaf color, especially delphinidin-3-O-glucoside, a type of anthocyanin responsible for the red, purple, or blue colors in various plant tissues. Delphinidin is known to play a key role in purplish-red leaf formation in certain plant species, such as Formosan sweet gum (*Liquidambar formosana* Hance), which exhibits attractive autumnal leaf coloration [45]. Therefore, it is likely that delphinidin-3-O-glucoside contributes to the intense purplish-red color of the second leaf in EP2. Additionally, other pigments like chlorophyll in different leaves may also play a role in leaf color. As leaves mature, the content of some flavonoids like delphinidin-3-O-glucoside decreases, while chlorophyll content increases and gradually becomes the dominant pigment, causing the mature leaves to turn green.

Second leaves are typically located at the top of the plant canopy, making them more accessible to light exposure. However, they also have relatively immature photosystems [46], a reduced number and capacity of photosynthetic enzymes, and limited stomatal and cellular CO_2_ conductance. These factors make them more susceptible to photoinhibition. The higher abundance of anthocyanins, such as delphinidin-3-O-glucoside, in EP2 compared to EW2 in the second leaves might impact both leaf color and photoprotection. Anthocyanins, besides providing color to the leaves, also influence photosynthesis by acting as light screens, preventing the absorption of excess light energy, and protecting chloroplasts from photodamage [47]. Moreover, other flavonoids like astragalin, cyanidin 3-rutinoside, and kaempferol-3-O-rutinoside have strong antioxidant activity, and their abundance in EP2 could help protect leaves from oxidative stress caused by high light conditions [48, 49]. Therefore, the purplish-red color of EP2 could be an adaptive response to high light stress. However, the role of delphinidin-3-O-glucoside or other flavonoids like astragalin in photosystem protection requires further investigation. Future studies should focus on providing genetic and biochemical evidence to elucidate the mechanisms of these compounds in photoprotection.

Transcriptomic analysis provides insights into the key genes influencing the synthesis and accumulation of anthocyanins in leaves. In our study, *CHS* genes involved in the purple anthocyanin biosynthetic pathway were found to be up-regulated in EP compared to EW. These genes likely contribute to the higher accumulation of specific flavonoids, such as delphinidin-3-O-glucoside, astragalin, cyanidin 3-rutinoside, and kaempferol-3-O-rutinoside, in the purplish-red leaves of EP. Transcriptomic analyses have revealed in many species that *CHS* is an important regulatory point for the biosynthesis of purple anthocyanins, as observed in purple kiwifruit (*Actinidia* species) [50], pink flowers of anthocyanin-rich tea (*Camellia sinensis*) [51], and grape berry skins [52].

Regulation of anthocyanin biosynthesis primarily occurs at the gene expression level, involving regulatory and structural genes. The MBW complex is a well-studied regulatory element in anthocyanin biosynthesis, responding to various environmental factors like cold and different light conditions. Research indicates that the MBW complex directly interacts with the promoters of structural genes, typically promoting their expression. Other regulatory genes implicated in anthocyanin biosynthesis, such as HY5 [53], HYH [54], and MDAS-box genes [55], have been previously reported, but in our study, we did not detect those TF genes in DEGs between EP2 and EW2, suggesting a different regulatory mechanism under these environmental conditions. Additionally, while R2R3-MYB and bHLH regulatory genes often exhibit specific expression in pigmented tissues, WD40 genes, responsible for stabilizing the MBW complexes, generally show similar expression levels between tissues with and without anthocyanin pigmentation [56, 57]. In our investigation, we did not detect differential expression of WD40 genes between EP2 and EW2. Transgenic tobacco experiments have provided strong evidence that overexpression of R2R3-MYB TF (CsMYB6A) has the capability to stimulate the expression of genes associated with flavonoid biosynthesis, particularly *CHS* and *3GT*, resulting in purple leaves [58]. In our study, we have identified a substantial number of differentially expressed genes (DEGs) that encode transcription factors (TFs). Among them, five genes are classified within the MYB family, and an additional ten are categorized as MYB-related TFs. A MYB gene, sequence similar to Arabidopsis MYB73 previously known to respond to cold stress and regulate anthocyanin biosynthesis, has been identified. However, further investigation is necessary to understand its specific involvement in anthocyanin or flavonoid biosynthesis, particularly its regulatory interactions with *CHS*. Confirming its functions requires detailed characterization through genetic and biochemical evidence.

In conclusion, our study provides valuable insights into the metabolomic and transcriptomic changes associated with leaf color variation in mulberry. The identification of key metabolic pathways and genes may pave the way for future investigations into the molecular mechanisms governing leaf pigmentation. Our findings contribute to the broader understanding of the economic importance of mulberry and its multifaceted applications in various industries, including pharmaceuticals and healthcare.

## Materials and methods

### Plant materials

Two mulberry genotype plants (EP and EW; Fig. 1), which are progenies of the same parent, were grafted onto a shared stump outdoors. This grafting took place in the mulberry germplasm resource nursery at Southwest University in Chongqing in January 2006. Both were grown under the same environmental conditions, water, cut and fertilizer management regimes, and pest and pathogen control. The second and fifth leaves growing on the upper branch were collected from EP and EW of main branches, and three biological replicates were performed for photosynthetic parameters measurements. Samples were harvested and measured before 12:00 am on 20th April 2021. Half of each leaf sample was used to extract photosynthetic pigments, while the remaining leaves were snap-frozen in liquid nitrogen and stored at -80 °C for subsequent transcriptome sequencing and metabolite extraction. Three independent biological replicates were collected per treatment.

### Measurements of Chl a, Chl b, concentrations

Leaf tissue (0.1 g) from each genotype was immersed in 10 ml of a 80% acetone to extract Chl a, Chl b under − 20 °C. The absorbance of pigment extracts at 663, 645 and 700 nm were recorded using a spectrophotometer (Techcomp UV1000, Shanghai China). Chla and Chlb were calculated by the formula as follows: Chla (mg/ml) = 12.7*(A_663_-A_700_)-2.69*(A_645_-A_700_) and Chlb (mg/ml) = 22.9*(A_645_-A_700_)-4.68*(A_663_-A_700_). Measurements for the two genotypes of mulberry were repeated three times, and averages were used as the final result.

### Transcriptome analysis of mulberry leaf

Total RNA was extracted by TRIzol (Invitrogen, CA, United States), and RNA quality was assessed. Next, the RNA samples were subjected to library construction and sequencing in Beijing Allwegene Gene Technology Co., Ltd. using the Illumina Novaseq 6,000, PE 150 sequencing platform. STAR2 soffware (v2) aligned the clean data to the reference genome (Camellia sinensis genome GCF_004153795.1) [59]. Each sample’s gene expression level and differential expression were analyzed using HTSeq and DESeq soffware (1.10.1), respectively [60]. | log2 (Fold Change) |>1 and the q value cutoff criteria to screen the differentially expressed genes (DEGs) between LF and Control groups. TTe GOseq R package (v1.22) was used for the gene ontology (GO) enrichment analysis of the DEGs, and KOBAS soffware (v2.0) was used to analyze DEG enrichment in KEGG pathways [61].

### Measurements of photosynthetic parameters

The leaves underwent a 15-minute period of dark adaptation prior to the assessment of various photochemical parameters, including Fv/Fm, F0, Fm and NPQ. The chlorophyll florescence was measured using a handheld chlorophyll fluorometer (Zealquest Scientific Technology Co., Ltd FP110, Shanghai, China). Net photosynthesis rate, stomatal conductance, intercellular carbon dioxide concentration (Ci), and transpiration rate (E) were determined using an LCproT analyzer manufactured by Zealquest Scientific Technology Co., Ltd (Shanghai, China). Each experiment was conducted with a minimum of three repetitions. Measurements were taken on leaves approximately 2 cm away from the central leaf vein, near the midpoint.

### Non-target metabolome analysis of mulberry leaf

Tissue samples were subjected to metabolite extraction. 50 mg of samples were homogenized with 500 µl pre-cooled methanol: water (3:1) at 35 Hz for 4 min, then ultrasonic treated with ice water bath for 5 min. After centrifugation at 12,000 rpm at 4 °C for 15 min, 200 µl supernatant was evaporated in a vacuum concentrator, and 40 µl of Methoxyamination hydrochloride (20 mg/ml in pyridine) was added and then incubated at 80 °C for 30 min, then derivatized by 60 µl of BSTFA regent (1% TMCS, v/v) at 70 °C for 1.5 h. After gradually cooling to the room temperature, 5 µl of FAMEs (in chloroform) was added. Metabolomics data analysis was performed using gas chromatography quadrupole time of flight mass spectrometry technology (GC-QTOF-MS, Agilent 7,890) equipped with a DB-5MS capillary column (30 m × 250 μm × 0.25 μm, J&W Scientiffc, Folsom, CA, United States). Further analysis of metabolites was assisted by Beijing Allwegene Gene Technology Co., Ltd. The relationship between metabolite expression and sample category was modeled using orthogonal partial least squares discriminant analysis (OPLS-DA) regression to achieve sample category modeling predictions (MetaboAnalystR-3.6.0) [62]. GC-QTOF-MS metabolomic data were analyzed through multivariate statistical methods (MetaboAnalystR-3.6.0). Card value standards were set for screening differential metabolites: the Student’s t-test p-value was less than 0.05, and the variable importance of projection in the OPLS-DA model’s first principal component was greater than 1. Subsequently, differential metabolites were projected to authoritative metabolite databases, such as KEGG (https://www.kegg.jp/) and (MetaboAnalyst https://www.metaboanalyst.ca/) to identify involved pathways. Further metabolic pathway analysis on the differential metabolites examined the correlation between metabolic pathways and biological issues.

### Electronic supplementary material

Below is the link to the electronic supplementary material.

**Supplementary Material 1: Fig S1.** Parameters involved in photosynthesis in the second leave of EP and EW. a, Rate of photosynthesis in leaves. b, Intercellular CO2 in leaves. c, Stomatal conductance in leaves. d, Transpiration rate in leaves. Statistical significance within the figure is denoted by alphabetical markers (a, b, c, d). The experiments were conducted in triplicate

**Supplementary Material 2: Fig S2.** Phylogenetic tree analysis (A) and sequence similarity (B) of malb00000676 with its homologs in Arabidopsis and poplar. ATG2G23290, AT3G55730, AT2G39880, AT4G37260 are from Arabidopsis; KAJ693982 from poplar

**Supplementary Material 3: Fig S3.** Phylogenetic tree analysis and sequence similarity of malb00000833 with its homologs in Arabidopsis and apple (Malus domestica). NP 178199, NP199763.1, NP001331284.1, OAP14833, OAO98848.1 and AAK28312.1 are from Arabidopsis; XP017189732.1 from poplar

**Supplementary Material 4: Fig S4.** Class analysis of differential expressed in EP2 verse EW2 that function associated with flavonoid biosynthesis

**Supplementary Material 5: Table S1**. All components detected by metabolic analysis. ID, The unique data number of this substance in this qualitative analysis; KEGG_ID, Substance number in KEGG database; EXACT_MASS, Exact molecular weight; NAME_EN, English name of substance; NAME_CH, Chinese name of substance; CLASS_EN, Substance Classification-English; CLASS_CH, Substance Classification-Chinese; ion mode positive and negative ion mode, - is negative, + is positive; rt, sample quantitative value; QC, Quality control samples, After the sample extraction, all samples of equal volume are taken and then mixed

**Supplementary Material 6: Table S2**. Gene lists used for venn plot in Fig 5

**Supplementary Material 7: Table S3**. Transcription factors identified in defferential expressed genes in EP2 compared to EW2 using plant trancription factor database V4.0 (http://planttfdb.gao-lab.org/)

**Supplementary Material 8: Table S4**. Flavonoid biosynthesis DEGs in EP2/EW2 identified by mapman annotation (https://mapman.gabipd.org/mapman)

## Data Availability

All data sheets and codes to process data are available upon request to the corresponding author, Xiling Wang (wxlswu@163.com). Sequence data generated for this study is available publicly in the NCBI Sequence Read Archive under BioProject ID: PRJNA1020653 (https://www.ncbi.nlm.nih.gov/).

## Abbreviations

CHS: chalcone synthase
CHI: chalcone isomerase
F3H: flavanone 3-hydroxylase
F3’H: flavonoid 3’-hydroxylase
DFR: dihydroflavonol 4-reductase
ANS: anthocyanidin synthase
EW: wild-type like
EP: purplished-red young leaves
LC-MS/MS: liquid chromatography tandem mass spectrometry
DEMs: differentially expressed metabolites
DEGs: differentially expressed genes
