# Supplementary figures and images for: Molecular and metabolic insights into purplish leaf coloration through the investigation of two mulberry (Morus alba) genotypes
Source: BMC Plant Biol. 2024 Jan 23;24:61. doi: 10.1186/s12870-024-04737-x (PMC10804552; doi:10.1186/s12870-024-04737-x)

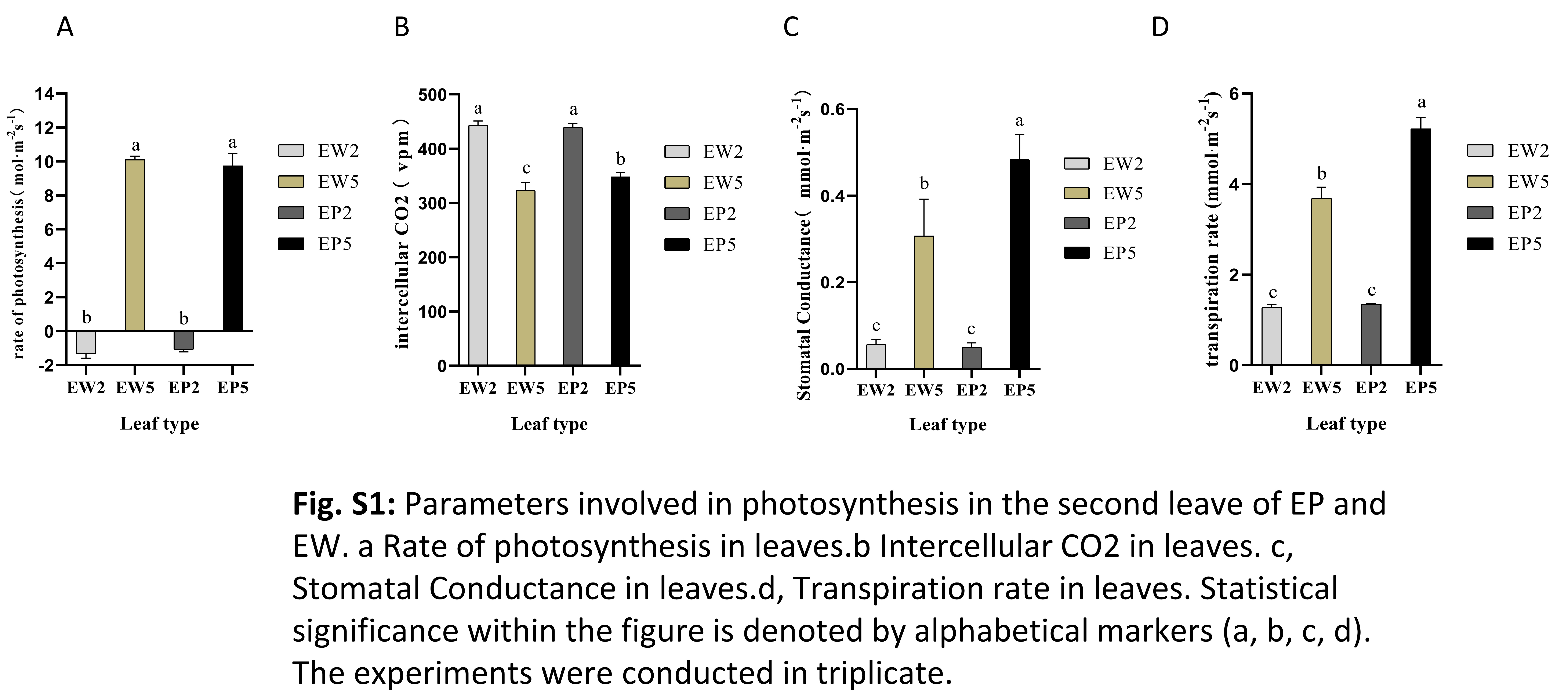

Supplement: Supplementary file 1 — Supplementary Material 1: Fig S1. Parameters involved in photosynthesis in the second leave of EP and EW. a, Rate of photosynthesis in leaves. b, Intercellular CO2 in leaves. c, Stomatal conductance in leaves. d, Transpiration rate in leaves. Statistical significance within the figure is denoted by alphabetical markers (a, b, c, d). The experiments were conducted in triplicate [file 12870_2024_4737_MOESM1_ESM.tif]
